# The Self-Assessment Scale of Cognitive Complaints in Schizophrenia: A validation study in Tunisian population

**DOI:** 10.1186/1471-244X-9-66

**Published:** 2009-10-08

**Authors:** Ines Johnson, Oussama Kebir, Olfa Ben Azouz, Lamia Dellagi, Yasmine Rabah, Karim Tabbane

**Affiliations:** 1 Research Unit "Cognitive dysfunctions in psychiatric diseases", Department of psychiatry "B", Razi Hospital. 24, rue des orangers. La Manouba, Tunisia

## Abstract

**Background:**

Despite a huge well-documented literature on cognitive deficits in schizophrenia, little is known about the own perception of patients regarding their cognitive functioning. The purpose of our study was to create a scale to collect subjective cognitive complaints of patients suffering from schizophrenia with Tunisian Arabic dialect as mother tongue and to proceed to a validation study of this scale.

**Methods:**

The authors constructed the Self-Assessment Scale of Cognitive Complaints in Schizophrenia (SASCCS) based on a questionnaire covering five cognitive domains which are the most frequently reported in the literature to be impaired in schizophrenia. The scale consisted of 21 likert-type questions dealing with memory, attention, executive functions, language and praxia. In a second time, the authors proceeded to the study of psychometric qualities of the scale among 105 patients suffering from schizophrenia spectrum disorders (based on DSM- IV criteria). Patients were evaluated using the Positive and Negative Syndrome Scale (PANSS), the Global Assessment Functioning Scale (GAF scale) and the Calgary Depression Scale (CDS).

**Results:**

The scale's reliability was proven to be good through Cronbach alpha coefficient equal to 0.85 and showing its good internal consistency. The intra-class correlation coefficient at 11 weeks was equal to 0.77 suggesting a good stability over time. Principal component analysis with Oblimin rotation was performed and yielded to six factors accounting for 58.28% of the total variance of the scale.

**Conclusion:**

Given the good psychometric properties that have been revealed in this study, the SASCCS seems to be reliable to measure schizophrenic patients' perception of their own cognitive impairment. This kind of evaluation can't substitute for objective measures of cognitive performances in schizophrenia. The purpose of such an evaluation is to permit to the patient to express his own well-being and satisfaction of quality of life.

## Background

It is now well proven that schizophrenia is associated with multiple cognitive deficits [1-3] which can be profound and devastating [4]. Patients with chronic schizophrenia demonstrate impairments that range between one and a half to two standard deviations below healthy controls on several key dimensions of cognition [5], especially verbal memory, working memory, motor speed, attention, executive functions and verbal fluency [6].

These deficits are thought to be a core feature of schizophrenia and not simply the result of the symptoms or the current treatments of the illness [7,8]. Moreover, they seem to have an impact on functional outcome [9] as they are correlated with poor functional abilities including skills acquisition, problem solving, and community living [10-12]. Furthermore, neurocognitive deficits are believed to be the single strongest correlate of real-world functioning [13].

The number of publications on cognitive deficits in schizophrenia has grown vastly over the past two decades. At the same time, an increasing number of sophisticated laboratory tasks has been developed for a better assessment of cognition [14]. However, little is known about how patients suffering from schizophrenia perceive their own cognition. Are they aware of their eventual cognitive impairments? Do they realize that their social functioning is highly influenced by these deteriorations? Do they complain about their memory problems to their doctor and do they demand specific treatments for them?

Traditionally, the study of subjective symptoms of schizophrenic patients has been limited to delusions and hallucinations [15]. Nowadays, abnormal subjective experiences concerning fields other than delusions and hallucinations are becoming more investigated since they are believed to be important in understanding and treating schizophrenia [16,17]. From a historical point of view, the first author who described a patient's subjective experiences in schizophrenia was Huber [18,19]. This German author introduced the term of "basic symptoms" to designate the first symptoms of schizophrenia that constitute the basis on which the others symptoms develop. These symptoms do not include behavioural abnormalities or verbal impairments that can be assessed objectively by clinicians. In fact, they are only reported by patients that describe them as subjective experiences of deficits including loss of energy, motor dysfunctions, abnormal corporeal sensations, altered cognitive processes, difficulties to feel emotions and vulnerability to stress [20]. The basic symptoms were targeted by a multitude of scales comprising the Bonn Scale [21], the Frankfurt Complaint Scale [22], the Subjective Experience of Deficit Scale [23], the Interview on Subjective Experience [16], the Subjective Deficit Syndrome Scale [24] and the Ependorff Inventory of Schizophrenia [25]. What is significant is that these scales dealt with different aspects of subjective experiences in schizophrenia including cognitive dysfunctions but didn't focus specifically on the latter. Only one scale, the SSTICS or Subjective Scale To Investigate Cognition in Schizophrenia [14], assessed specifically the cognitive subjective symptoms in schizophrenia. The psychometric properties of this scale were evaluated within a population of 114 French speaking schizophrenic patients. Validation study of the SSTICS was shown to be successful proving that cognitive complaints in schizophrenia can be reliably assessed.

To our knowledge, no similar instrument has been published and validated in the Arabic language. Consequently, the purpose of our study was to create a scale to collect subjective cognitive complaints of patients suffering from schizophrenia whose mother tongue is Tunisian Arabic.

## Methods

### Description of the scale

The authors constructed the Self-Assessment Scale of Cognitive Complaints in Schizophrenia (SASCCS) based on a questionnaire covering five cognitive domains which are the most frequently reported in the literature to be impaired in schizophrenia [6,26]. The scale consisted of 21 questions dealing with memory, attention, executive functions, language and praxia. Memory was evaluated through its components: working memory (item 1&2), episodic memory (item 3 though 9) and semantic memory (item 10&11). Attention was investigated through its components: distractibility (item 12), alertness (item13), selective attention (item14), divided attention (item15) and sustained attention (item16). Executive functions were explored through their components: planning (item17), organisation (item18) and flexibility (item19). Finally, language was examined through item 20 and praxia through item 21. The scale was made to be as clear, simple and easy to use by patients suffering from schizophrenia. It was written in Tunisian Arabic dialect. 'See additional file 1: Tunisian version of the SASCCS'. 'See additional file 2: English version of the SASCSS'.

### Pre-test of experimental version

The questionnaire was first administered to a reduced sample of 38 patients (35 men, 3 women) meeting the DSM-IV diagnostic criteria for schizophrenia (n = 35) or schizoaffective disorder (n = 3) [27]. The aim of this preliminary work was to collect comments from both patients and investigators in order to better formulate the items and furthermore, to add examples to the questions that closely suit the patient's daily life. Mean age of the patients was 34 ± 8.9 years and time elapsed since onset of the disease was 10.3 years (SD = 6.89). 'See additional file 3: Table S1: demographic characteristics and psychiatry history of pre-test sample'.

Mean total score of the PANSS was 61 ± 16 [28].

Accordingly to this purpose, item 8 was modified in a way to provide examples corresponding to both men and women in their daily activities. The wording of items 10, 14 and 15 was reviewed in a way to be clarified. This pre-test also served to harmonize the modalities of the scale's administration and the instructions given each time to the patients.

### Administration procedure

The SASCCS is a self-rated questionnaire administered during a structured interview in which the investigator explains to the patient the way he should answer to the 21 Likert-type questions of the scale. The patient is asked to read each of the items in which problems of memory or concentration of daily life are presented and may have been experienced by him self. He is then asked to estimate the frequency of occurrence of such situations in his own life. For that purpose, he must circle the number that best corresponds to his experienced life. (4-very often; 3-often; 2-sometimes; 1-rarely; 0-never). The SASCCS total score is calculated by adding each item score together. The more the patient complained about cognitive impairments, the higher was the scale's total score.

The approximate time to completion was 15 minutes on average. The questionnaire was administered at the outpatient clinic. The same trained psychiatrist proceeded to the administration of the scale among all participants. The investigator should remain on site until the patient is done with the questionnaire. He could provide explanations to some questions or even examples to clarify the meaning of items especially item 13, 15, 18 and 19. 'See additional file 4: examples for items 13, 15, 18 and 19 of the SASCCS'.

### Characteristics of the population

The final version of the scale was then administered to 105 outpatients who met the DSM IV criteria for schizophrenia (undifferentiated subtype, n = 47; paranoid subtype, n = 39; hebephrenic subtype, n = 6; residual subtype, n = 3) or schizoaffective disorder (bipolar subtype, n = 8; depressive subtype, n = 2). Patients were recruited from three different outpatient clinics based in the Razi Hospital (La Manouba, Tunisia). They were carefully screened to rule out an additional Axis I diagnosis or any disorder that might alter brain functioning. They had to meet the following requirements:

(1) have a minimum educational level of 5 years,

(2) no evidence of mental retardation,

(3) being at the time of testing under unchanged medication dosage for the last 4 weeks.

(4) never undergone electroconvulsive therapy,

(5) no evidence of organic brain pathology including cerebral tumor, epilepsy, systemic disease, history of cranial trauma, brain surgery

(6) no history of substance abuse or dependence, and consumption of psychoactive.

Table 1 shows sociodemographic sample characteristics and its psychiatric history.

**Table 1:** Demographic sample characteristics and psychiatric history

**Variable**		
Age (years; mean, SD)	34	7
Gender (n)		
Male	86	81.9%
Female	19	18.1%
Years of education (mean,SD)		
	9.7	3.1
Marital status (n)		
Single	91	86.7%
Married	10	9.5%
Divorced	4	3.8%
Occupation (n)		
Unemployed	60	57.2%
Working	40	38.1%
Studying	3	2.9%
Retired	2	1.9%
Duration of illness (years; mean, SD)	10.17	6.01
Number of hospitalisations (mean, min-max)	3	[0-22]
Total period of hospital stay (weeks; mean, min-max)	10.38	[0-60]
Neuroleptics (n)		
Neuroleptics (n) First generation	76	72.4%
Second generation	29	27.6%
Chlorpromazine equivalent of antipsychotic dosage (mean, SD)	482.5	322

### Psychopathological assessment

Psychopathological symptoms were evaluated using the PANSS [29], the Calgary depression scale (CDS) [30] and the Global Assessment Functioning scale (GAF scale) [27]. PANSS, CDS and EGF were administered by the same trained psychiatrist for all participants. Mean scores on these clinical scales were as follows: 52.84 (SD = 9.64) for the PANSS total score, 1.35 [min = 0; max = 5] for the CDS and 62.58 (SD = 13.88) for the GAF scale. Mean scores for the PANSS subscales were as follows: 10.05 (SD = 2.5) for the positive symptoms, 16.32 (SD = 4.49) for the negative symptoms and 26.4 (SD = 5) for general psychopathology. Mean score for the item G12 of the PANSS assessing insight was 2.32 (SD = 1.15).

Using the 5-factor model of the PANSS as identified by Lindenmayer et al. [31], we calculated the cognitive factor and the depression factor which had respectively a score of 10.14 (SD = 2.49) and 5.93 (SD = 1.99).

### Statistical analysis

We conducted an exploratory principal component analysis (PCA) on the correlation matrix of the 21 items of the SASCCS. Several guidelines were used to select the number of factors: the Kaiser criteria and the interpretability of the factors. Oblimin rotation was then performed.

Construct validity and reliability were evaluated by calculating Cronbach's alpha coefficient and the average of correlations between each item and the total score.

Correlation analyses were performed using the Pearson coefficient when data had normal distribution; elsewhere, Spearman rank correlation was calculated.

Statistical significance level was set at *p = 0.01 (*two-tailed).

Statistical analyses were performed using SPSS software in his 12^th ^version.

### Ethics and Consent

This research has been undergone in a psychiatric university department in RAZI hospital. It has been approved by the local ethic committee. Patients have signed a written and informed consent.

## Results

The SASCCS global score mean was 24.98 (SD = 14.83; min = 0, max = 109; median = 24).

### Reliability

#### Internal consistency

It was evaluated by calculating Cronbach's alpha coefficient [32] which was equal to 0.85 proving a good internal consistency of the scale but furthermore, a satisfactory reliability of its measure.

#### Test-retest reliability

Its was assessed within a subgroup of 39 patients examined by the same investigator at a mean interval of 80 days (SD = 33). Intra-class correlation coefficient was equal to 0.77 (*p *= 0.00) suggesting a good stability over time.

### Validity of internal structure

We carried out a factor analysis using principal component analysis as the extraction method. The decision-making for factor extraction was based on Kaiser criteria [33]. According to these criteria, the factors extracted should have an eigenvalue greater than 1, provided that the total variance explained exceeded 50%. PCA with Oblimin rotation yielded six factors with 58.2% explained variance (Table 2). The eigenvalues of the first two factors were 5.57 and 1.61, respectively, and the corresponding variances were 26.55% and 7.68%.

**Table 2:** Principal component analysis: Total variance explained

**Factor**	**% of variance**	**Cumulative %**
1	26.55	26.55
2	7.68	34.24
3	6.77	41.01
4	6.55	47.56
5	5.65	53.22
6	5.06	58.28

In order to evaluate cognition as conceptualized by subjectivity, PCA with Oblimin rotation method [34] was performed to see whether latent variables would emerge and lead to a cognitive model different from the initial theoretical one that have been the basis of our scale. After carrying out an Oblimin Rotation, the items with a loading higher then 0.50 were retained to be part of the subjective cognitive factors (Table 3).

**Table 3:** Subjective cognitive domains of complaints

**Subjective Factors**	**Items**	**Subjective domains**
1	5, 12, 16, 20, 21	Distractibility
2	8, 9, 15, 18	Daily life
3	10, 11	Semantic memory
4	4, 6, 7	Disorder consciousness
5	1, 2	Working memory
6	3, 14, 17, 19	Executive skills

### Correlations between psychopathological assessment and scale's scores

We examined whether correlations existed between scores derived from the scale and positive, negative and disorganisation factors derived from the factorial analyses studies of the PANSS [35]. We also considered the item G 12 assessing insight as well as the Calgary Depression Scale total score.

The SASCCS total score wasn't correlated to any of the PANSS. A weak negative correlation between the SASCCS total score and PANSS insight score was found (*r *= -.21) but didn't reach the statistical significance (*p *= .03). Calgary score was correlated with the SASCCS total score (*r *= .33; *p *= .001).

The cognitive factor of the 5-factor model of the PANSS wasn't correlated to the SASCCS total score or sub-scores.

The depression factor was correlated to the SASCCS total score (*r = .20*) although this correlation didn't reach the statistical significance (*p = .03*).

## Discussion

The aim of this study was to construct and to validate a scale to measure the subjectivity of patients with schizophrenia regarding their cognition. The SASCCS, which was easy to administer in less than 15 minutes, had good reliability and stability over time. No cut-off has been determined for this scale. In fact, the SASCCS total score is used to estimate a patient's level of complaining.

The composition of subjective cognitive domains as derived from factor analysis was slightly different from that of the initial theoretical model which has been the basis of the scale's construction. Actually, the scale's items have been distributed after PCA differently from the original structure of the scale leading to a neo-construct of the instrument. These differences were not surprising since the questionnaire was based on the neuropsychological theoretical conception of cognition whereas factor analysis of the scale reflected the patient's own perception of his cognition. Stip et al., using the Subjective Scale To Investigate Cognition in Schizophrenia (SSTICS), have also found a difference between the distribution of the items in the initial model and in the neo-construct of their scale [14]. It could be that the selected items did not exactly measure what they were supposed to. Also, their specificity might be imperfect as it refers to several overlapping dimensions.

These findings point to the complex representation of schizophrenic patients of their own cognition. And even though the latter does not correspond to the theoretical construct of cognition, the scale remains reliable because of both its good internal consistency and stability over time.

During this study, no other instrument evaluating cognitive functions was administered simultaneously to our population. Therefore, convergent validity was unnecessary. However, when reviewing the literature, no positive correlation was found between objective and subjective scores of cognition. Using the SSTICS, Prouteau et al. found that cognitive nature of subjective complaints did not strictly match with that of impaired objective performances [36]. Chan et al. assessed prospective memory in patients with schizophrenia and did not find a correlation between objective performances and subjective measures of this cognitive function [37].

These results suggest that subjective evaluation of cognition could be an independent dimension from its objective assessment in patients with schizophrenia.

In fact, in our study, no correlation has been found between the SASCCS scores and the PANSS cognitive factor which could also point to the fact that self-assessment of cognition is a totally independent aspect from clinical evaluation of the cognitive functions.

The correlation of insight with subjective perception of cognition in schizophrenia is an aspect that deserves to be considered and analyzed.

In fact, awareness of one's own cognitive deficits could be highly influenced by consciousness of one's whole condition as a mentally ill person. Schizophrenia is generally accompanied by a lack of insight meaning an impaired awareness of one's psychiatric condition and life situation [38]. Therefore, low scoring at the SASCCS could be due to a lack of insight.

In our study, a weak negative correlation between PANSS insight score and SASCCS score has been found (*r *= -.21, *p = .03*) but didn't reach the significance level set at 0.01. However, it should be noticed that our study included a majority of subjects scoring no more than 4 on the PANSS insight item. Only one patient had a score of 5.

Since insight could influence one's subjective perception of cognition, it is recommended to evaluate patient's insight while using the SASCCS.

Another important factor to be considered is depression since a depressive state could be accompanied by cognitive disturbances in several domains such memory and attention [39,40].

In our study, there was a positive correlation between SASCCS total score and CDS score meaning that the more depressive symptomatology is severe, the more the patient reports cognitive troubles. Although it was not statistically significant, we also did find a correlation between SASCCS total score and the depression score of the 5-factor model of the PANSS suggesting the influence that could exert depression on self assessment of cognition by emphasizing cognitive complaints when being more depressed.

Lecardeur et al. found in their study using the SSTICS a correlation between the scale total score and the PANSS depression score. It could be suggested that subjective complaints of cognitive deficits may influence a patient's objective depressive state as rated by the clinician [41].

Considering the influence of depressive traits on subjective perception toward cognition, we recommend measuring the patient's mood state when using the SASCCS.

## Conclusion

We present here a self-assessment scale to evaluate cognitive deficits as perceived by patients suffering from schizophrenia in domains of memory, attention and executive functions. Given the good psychometric properties that have been revealed in this study, the SASCCS seems to be reliable to measure schizophrenic patients' perception of their own cognitive impairment. This kind of evaluation can not replace objective measures of cognitive performances in schizophrenia. Actually, the purpose of such an evaluation is to allow the patient to express his own well-being and satisfaction of quality of life. Furthermore, subjective evaluation of cognitive functions could provide a more complete picture of the cognitive profile of an individual. Therefore, better therapeutic targets could be adapted to his condition during cognitive rehabilitation programs.

## List of abbreviations

SASCCS: Self-Assessment Scale of Cognitive Complaints in Schizophrenia; PCA: Principal Component Analysis; SSTICS: Subjective Scale To Investigate Cognition in Schizophrenia.

## Competing interests

The authors declare that they have no competing interests.

## Authors' contributions

IJ, OK and OBA led the study concept and design, data collection, data analysis, and drafting of the manuscript. LD, YR and KT participated in the pre-test of experimental version of the scale. LD and YR participated in data collection. All authors read and approved the final manuscript.

## Pre-publication history

The pre-publication history for this paper can be accessed here:



## Supplementary Material

Additional file 1 **Tunisian version of the SACSS**. this is the original version of the SASCCS scale written in Tunisian Arabic. Click here for file

Additional file 2 **English version of the SACSS (not validated)**. this is the English version of the SASCCS which is not validated. Click here for file

Additional file 3 **Table S1: demographic characteristics and psychiatry history of pre-test sample**. this table describes the sociodemographic characteristics of pre-test sample as well as its psychiatric history. Click here for file

Additional file 4 **examples for items 13, 15, 18 and 19 of the SASCCS**. these are the examples that the investigator could provide to the patient when administering the SASCCS to clarify the meaning of items 13, 15, 18 and 19. Click here for file

## References

[B1] Heinrichs RW, Zakzanis KK (1998). Neurocognitive deficits in schizophrenia: A quantitative review of the evidence. Neuropsychol.

[B2] Fioravanti M, Carlone O, Vitale B, Cinti ME, Clare L (2005). A meta-analysis of cognitive deficits in adults with diagnosis of schizophrenia. Neuropsychol Rev.

[B3] Keefe RSE, Fenton WS (2007). How Should DSM-V Criteria for Schizophrenia Include Cognitive Impairment?. Schizophr Bull.

[B4] Green MF, Kern RS, Braff DL, Mintz J (2000). Neurocognitive deficits and functional outcome in schizophrenia: are measuring the « right stuff?? ». Schizophr Bull.

[B5] Keefe RS (2008). Should cognitive impairment be included in the diagnostic criteria for schizophrenia?. World Psychiatry.

[B6] Keefe RS, Goldberg TE, Harvey PD, Gold JM, Poe MP, Coughenour L (2004). The Brief Assessment of cognition in schizophrenia: reliability, sensitivity and comparison with a standard neuroccognitive battery. Schizophr Res.

[B7] Green MF, Kern RS, Heaton RK (2004). Longitudinal studies of cognition and functional outcome in schizophrenia: implications for MATRICS. Schizophr Res.

[B8] Saykin AJ, Shtasel DL, Gur RE, Kester DB, Mozley LH, Stafiniak P (1994). Neuropsychologial deficits in neuroleptic naïve patients with first-episode schizophrenia. Arch Gen Psychiatry.

[B9] Green MF, Kern RS, Braff DL, Mintz J (2000). Neurocognitive deficits and functional outcome in schizophrenia: are we measuring the « right stuff?? ». Schizophr Bull.

[B10] Dickinson D, Bellack AS, Gold JM (2007). Social/Communication Skills, Cognition, and Vocational Functioning in Schizophrenia. Schizophr Bull.

[B11] McClure MM, Bowie CR, Patterson TL, Heaton RK (2007). Correlations of functional capacity and neuropsychological performance in older patients with schizophrenia: Evidence for specificity of relationships?. Schizophr Res.

[B12] Cohen AS, Forbes CB, Mann MC, Blanchard JJ (2006). Specific cognitive deficits and differential domains of social functioning impairment in schizophrenia. Schizophr Res.

[B13] Green MF (1996). What are the functional consequences of neurocognitive deficits in schizophrenia?. Am J Psychiatry.

[B14] Stip E, Caron J, Renaud S, Pampoulova T, Lecompte Y (2003). Exploring cognitive complaints in schizophrenia: The Subjective Scale To Investigate cognition in Scizophrenia. Compr Psychiatry.

[B15] Peralta V, Cuesta MJ (1994). Subjective experience in schizophrenia: A critical review. Compr Psychiatry.

[B16] Cutting J, Dunne F (1989). Subjective experience of schizophrenia. Schizophr Bull.

[B17] Strauss JS (1989). Subjective experiences of schizophrenia: toward a new dynamic psychiatry-II. Schizophr Bull.

[B18] Huber G (1957). Die coenesthetische Schizophrenie. Fortschr Neurol Psychiatr.

[B19] Huber G (1966). Reine Defektsyndrome und Basisstadien endogener Psychosen. Fortschr Neurol Psychiatr.

[B20] Koehler K, Saller H (1984). Huber's Basic symptoms: Another Approach to Negative Psychopathology in schizophrenia. Compr Psychiatry.

[B21] Gross G, Huber G, Klosterkotter J, Linz M (1987). Bonn Scale for Assassment of Basic Symptoms.

[B22] Sullwold L (1986). Frankfurter Beschwerde-Fragboden (FBF). Schizohrene Basisstorungen.

[B23] Liddle PF, Barnes TR (1988). The subjective experience of deficits in schizophrenia. Compr Psychiatry.

[B24] Jaeger J, Bitter I, Czobor P, Volavka J (1990). The measurement of subjective experience in schizophrenia: the Subjective Deficit syndrome scale. Compr Psychiatry.

[B25] Mass R (2000). Characteristic subjective experiences of schizophrenia. Schizophr Bull.

[B26] Saykin AJ, Gur RC, Gur RE, Mozley PD, Mozley LH, Resnick SM, Kester DB, Stafiniak P (1991). Neuropsychological function in schizophrenia. Selective impairment in memory and learning. Arch Gen Psychiatry.

[B27] American Psychiatric Association (1994). Diagnostic and Statistical Manual of Mental Disorders.

[B28] Johnson I, Dhouib S, Bouaziz N, Ketata W, Dellagi L, Kebir O, Ben Azouz O, Tabbane K (2007). Evaluation de la perception subjective des déficits cognitifs chez les patients atteints de schizophrénie [Abstract]. L'Encéphale.

[B29] Kay SR, Fiszbein A, Opler LA (1987). The positive and negative syndrome scale (PANSS) for schizophrenia. Schizophr Bull.

[B30] Addington D, Addington J, Maticka-Tyndale E (1994). Specificity of the Calgary Depression Scale for schizophrenics. Schizophr Res.

[B31] Lindenmayer JP, Bernstein-Hyman R, Grochowski S (1994). Five-factor model of schizophrenia. Initial validation. J Nerv Ment Dis.

[B32] Cronbach LJ (1951). Coefficient alpha and the internal structure of tests. Psychometrica.

[B33] Jennrich RI, Sampson PF (1966). Rotation for simple loadings. Psychometrica.

[B34] Kaiser HF (1963). Image analysis.

[B35] Lepine JP, Piron JJ, Chapatot E, Stefanis CN, Soltados CR, Rabavilas AD (1989). Factor analysis of the PANSS in schizophrenic patients. Psychiatry today: accomplishments and promises.

[B36] Prouteau A, Verdoux H, Briand C, Lesage A, Lalonde P, Nicole L, Reinharz D, Stip E (2004). Self-assessed cognitive dysfunction and objective performance in out-patients with schizophrenia participating in a rehabilitation program. Schizophr Res.

[B37] Chan RCK, Wang Y, Ma Z, Chan RC, Wang Y, Ma Z, Hong XH, Yuan Y, Yu X, Li Z, Shum D, Gong QY (2008). Objective measures of prospective memory do not correlate with subjective complaints in schizophrenia. Schizophr Res.

[B38] Pini S, Cassano GB, Dell'Osso L, Amador XF (2001). Insight into illness in schizophrenia, schizoaffective disorder, and mood disorders with psychotic features. Am J Psychaitry.

[B39] Brébion G, Smith MJ, Amador X, Malaspina D, Gorman JM (1997). Clinical correlates of memory in schizophrenia: Differentail links between depression, positive and negative symptoms, and two types of memory impairment. Am J Psychiatry.

[B40] Holthausen EA, Wiersma D, Knegtering RH, Bosch RJ Van den (1999). Psychopathology and cognition in schizophrenic spectrum disorders: the role of depressive symptoms. Schizophr Res.

[B41] Lecardeur L, Briand C, Prouteau A, Lalonde P, Nicole L, Lesage A, Stip E (2009). Preserved awareness of their cognitive deficits in patients with schizophrenia: Convergent validity of the SSTICS. Schizophr Res.

