# Supplementary material for: The Self-Assessment Scale of Cognitive Complaints in Schizophrenia: A validation study in Tunisian population
Source: BMC Psychiatry. 2009 Oct 8;9:66. doi: 10.1186/1471-244X-9-66 (PMC2766383; doi:10.1186/1471-244X-9-66)
Supplement: Additional file 1 — Tunisian version of the SACSS. this is the original version of the SASCCS scale written in Tunisian Arabic. [file 1471-244X-9-66-S1.DOC]

**Échelle subjective d’évaluation des plaintes cognitives dans la schizophrénie**

**التّعليمات**

هاذوما مجموعة متاع أسئلة مطروح فيهم بعض مشاكل مَتَاعْ ذاكرة أو تركيز يُمكن قد تعرّضْتلهُم في حياتك اليوميّة . المطلوب منّك أنّك تحاول تحدّد نسبة المرّات إلّي عشت فيها هذه المشاكل. بمعنى آخر، كان ترى أنّ الوضعيّة المطروحة في السّؤال تعّرضتلها من قبل، آختار الإجابة إلّي تناسبك أحسن وحدة بين الآحتمالات هاذم:

- 4 بكثرة كبيرة
- 3 كثرة
- 2 ساعات
- 1 قليل

و كان ترى إلّي السّؤال ما يخصّكش، آختار الإجابة:

- 0 حتى مرة

1- ِتِحِسّشْ رُوِحكْ عَنِْدكْ مَشَاِكلْ مَتَاعْ ذاكرة ؟

- 4 بكثرة كبيرة
- 3 بكثرة
- 2 ساعات
- 1 قليل
- 0 حتى مرة

2- عَنِْدكْش صعوبة باش تتذكّر مَعْلومات عَرَفتها **جديدة** ِوِلّي ِيِِلزَِمكْ ِتسْتََحِْضرْهَا **وَقْتَها** ؟ مثلا: نُومْرُو ِتِليفُونْ، عُنْوَانْ، نُومْرُو كَارْ، وَإلآ إسْم طبيب ؟

- 4 بكثرة كبيرة
- 3 بكثرة
- 2 ساعات
- 1 قليل
- 0 حتى مرة

3- ِتلْقَاشْ صعوبة باش ِتِشّدْ حاجة في ذَاِكرِْتكْ ؟ مثلا: قائمة متاع قضْيَات المَرِْشي و إلآ قائمة متاع أسَاِمي عبَاد ؟

- 4 بكثرة كبيرة
- 3 بكثرة
- 2 ساعات
- 1 قليل
- 0 حتى مرة

4- ِتلقَاشْ صعوبة باش تتذكّر أسَاِمي دُواياِتكْ ؟

- 4 بكثرة كبيرة
- 3 بكثرة
- 2 ساعات
- 1 قليل
- 0 حتى مرة

5-عُمِْركش نَِسيتْ مَوِْعد مَعَ صَاحِْبكْ و إلآ مَوِْعد مَعَ طَِبيبْ ؟

- 4 بكثرة كبيرة
- 3 بكثرة
- 2 ساعات
- 1 قليل
- 0 حتى مرة

6- ِتنْسَاشْ باش تُشِْربْ دواياتك ؟

- 4 بكثرة كبيرة
- 3 بكثرة
- 2 ساعات
- 1 قليل
- 0 حتى مرة

7- ِتلقاشْ صعوبة باش تتذكّرأخْبَار قِريتْها في الجَريدَة و إلآ تفَرّجْت عَِليها في التّلفزة البارح؟

- 4 بكثرة كبيرة
- 3 بكثرة
- 2 ساعات
- 1 قليل
- 0 حتى مرة

8- عُمِْركش نِسيتْ ِكيفاش تطيّب مَاكلة مُعَيّنة وإلآ أشنُوّ البَهَارَاتْ إلٌي إيِِِجِيوْ ِفيها **/** وإلآ عُمِْركش نِسيتْ كيفاش تقوم ببعض التّصِْليحات في الدّار؟

- 4 بكثرة كبيرة
- 3 بكثرة
- 2 ساعات
- 1 قليل
- 0 حتى مرة

9- ِتلقاشْ صعوبة باش تَعِْرفْ ِكيفاشْ ِتمِْشي وَحِْدكْ للسِبيطَارْ و إلآ للمُسْتَوِْصفْ وإلآ حتّى لِدَاِركْ ؟

- 4 بكثرة كبيرة
- 3 بكثرة
- 2 ساعات
- 1 قليل
- 0 حتى مرة

10- ِتلقاشْ صعوبة باش تتذكّر أسَاِمي عباد تَاِبعَة المَجَالات إلٌي مَغْرُومْ بها ( الرياضة، السّينمَا ، الِغنَاء...)؟

- 4 بكثرة كبيرة
- 3 بكثرة
- 2 ساعات
- 1 قليل
- 0 حتى مرة

11- ِتلقاشْ صعوبة باش تتذكّر أسَاِمي أهَمّ المُدُنْ التّوِنِسيّة , وإلآ أحْدَاث هَامّة ِفي تَاِريخْ تونس ، وإلآ أسَامي البُلدَان الكُبْرَى؟

- 4 بكثرة كبيرة
- 3 كثرة
- 2 ساعات
- 1 قليل
- 0 حتى مرة

12- عَنِْدكش **السَّهْوَة** ؟ مثلا، وَقت إلٌي تَحِْكي مَعَ شَخْص و إلآ وَقت إلٌي تقْرَا ِفي جَِريدَة ؟

- 4 بكثرة كبيرة
- 3 كثرة
- 2 ساعات
- 1 قليل
- 0 حتى مرة

13 - ِتلقاشْ صعوبة في **الانِْتبَاه والتّصَرُّفْ ِبسُرْعَة** في **وَضِْعيّة ما كُنِْتشْ تِتْوَقّعْها** ؟ مثلا ، كَرْهَبَة صَادْمَة عَِليكْ وإنْتِ شاقّّ الكَيّاسْ

- 4 بكثرة كبيرة
- 3 كثرة
- 2 ساعات
- 1 قليل
- 0 حتى مرة

14- وَقْت إلٌي التلفزة مَحْلُولَة، الرّاديو ِيخِْدمْ، و فَمّا بَرْشَا عَباد يَحِْكيوْ بَحْذاكْ، ِتلقَاشْ صعوبة باش ترَِكّز على حاجة مُعَيّنَة؟

- 4 بكثرة كبيرة
- 3 كثرة
- 2 ساعات
- 1 قليل
- 0 حتى مرة

15- ِتلقاشْ صعوبة باش تَعِْملْ **حَاجِْتين في نَفس الوَقت** ؟ مثلا: تَحِْكي مَع وَاِحدْ وإنت ِتتفَِرّجْ على بَرْنَاِمجْ في التلفزة، و إلآ تَعِْمل في قَضِْية الدّار و ِتتفقّدْ فطُوِركْ ِمنْ وَقت إلى وَقت

- 4 بكثرة كبيرة
- 3 كثرة
- 2 ساعات
- 1 قليل
- 0 حتى مرة

16- ِتلقاشْ صعوبة باش تركّز على **نَفس الحَاجة أكِْثر ِمن 20 دقيقة** ؟ مثلا: تَسْمَعْ أخْبَارْ، وإلآ تقرَا جَِريدَة، وإلآ تتفَِرّجْ على مُسَلِسلْ، وإلآ تَحِْضرْ على دَرْس في القَسْم ؟

- 4 بكثرة كبيرة
- 3 كثرة
- 2 ساعات
- 1 قليل
- 0 حتى مرة

17- ِتلقاشْ صعوبة باش **تبَرّمِجْ أعْماِلك بالمّسبّق** ؟ مثلا، باش تجَِدّدْ كَرْنِي العِلاَجْ مَتَاِعكْ، وإلآ باش ِتتْعَدّى للبُوسْطَة باش تاِخذ الماندَة، وإلآ باش تقِسّمْ مَصْرُوِفكْ مَتاعْ الشّهَرْ؟

- 4 بكثرة كبيرة
- 3 كثرة
- 2 ساعات
- 1 قليل
- 0 حتى مرة

18- ِتلقاشْ صعوبة باش **تنّظم أعْماِلكْ اليوميّة** ؟ مثلا، القضية، الطبخ ، تنظيف الدّار، تصليح حاجات فيها، وإلآ الغسيل ؟

- 4 بكثرة كبيرة
- 3 كثرة
- 2 ساعات
- 1 قليل
- 0 حتى مرة

19- ِتلقاشْ صعوبة باش **تغَِيّرْ طَِريقَة تَفِْكيِِركْ أو طَِريقَة العَمَلْ إلّي ِمسْتاِنسْ بيها** وَقت إلّي ِيقتَرْحُوا عَِليكْ باش تغَيّرْهَا **وإنت مُوَاِفقْ عَلَى هَذا** ؟

- 4 بكثرة كبيرة
- 3 كثرة
- 2 ساعات
- 1 قليل
- 0 حتى مرة

20- عَنِْدكْش صعوبة باش **تَلْقَى كَلاِمكْ**، باش **تُكَِوّنْ جُمَلْ**، باش **ِتفِهمْ مَعْنَى كَِلمَاتْ** وإلآ باش ِ**تنْطَقهُم**، وإلآ باش **تُسَِمّي حاجات بآسمهم**؟

- 4 بكثرة كبيرة
- 3 كثرة
- 2 ساعات
- 1 قليل
- 0 حتى مرة

21- ِتلقاشْ صعوبة في **الِقيَامْ بأنِْشطَة عاِديّة** ؟ مثلا: ِكِتلِْبسْ و تَقِفلْ حُوَايِِْجكْ، ِكتدَِخّلْ ِمفتاح في كُوبَة، وإلآ ِكِتسْتَعِْملْ مغَرْفة ؟

- 4 بكثرة كبيرة
- 3 كثرة
- 2 ساعات
- 1 قليل
- 0 حتى مرة
