# Supplementary material for: The Self-Assessment Scale of Cognitive Complaints in Schizophrenia: A validation study in Tunisian population
Source: BMC Psychiatry. 2009 Oct 8;9:66. doi: 10.1186/1471-244X-9-66 (PMC2766383; doi:10.1186/1471-244X-9-66)
Supplement: Additional file 2 — English version of the SACSS (not validated). this is the English version of the SASCCS which is not validated. [file 1471-244X-9-66-S2.DOC]

Additional file 2: English version of the SASCCS (not validated)

1. Do you feel like you have memory disturbances?

- 4 Very often
- 3 Often
- 2 Sometimes
- 1 Few times
- 0 Never

1. Do you have any problems to remember information you’ve just learned and that you should immediately use? Example, an address, a telephone number, a bus number, a doctor’s name

- 4 Very often
- 3 Often
- 2 Sometimes
- 1 Few times
- 0 Never

1. Do you have difficulties to retain something in your mind? Example, a shopping list or a list of persons’ names

- 4 Very often
- 3 Often
- 2 Sometimes
- 1 Few times
- 0 Never

1. Do you have any problems to remember the name of your treatments?

- 4 Very often
- 3 Often
- 2 Sometimes
- 1 Few times
- 0 Never

1. Have you ever forgotten an appointment with your friend or with your doctor?

- 4 Very often
- 3 Often
- 2 Sometimes
- 1 Few times
- 0 Never

1. Do you sometimes forget to take your treatments?

- 4 Very often
- 3 Often
- 2 Sometimes
- 1 Few times
- 0 Never

1. Do you have any problems to remember information you learned in a paper or watched on TV yesterday?

- 4 Very often
- 3 Often
- 2 Sometimes
- 1 Few times
- 0 Never

1. Have you ever forgotten how to cook a dish or which ingredients you should put in / Have you ever forgotten how to fix or repair things at home

- 4 Very often
- 3 Often
- 2 Sometimes
- 1 Few times
- 0 Never

1. Do you have any problems to find your way by yourself to the hospital, the outpatient clinic or even to your home?

- 4 Very often
- 3 Often
- 2 Sometimes
- 1 Few times
- 0 Never

1. Do you have any problems remembering names of people belonging to fields you’re usually interested in? (sports, cinema, songs…)

- 4 Very often
- 3 Often
- 2 Sometimes
- 1 Few times
- 0 Never

1. Do you have any problems remembering names of the biggest towns in Tunisia or the most important historical events of your country, or the names of the biggest cities in the world?

- 4 Very often
- 3 Often
- 2 Sometimes
- 1 Few times
- 0 Never

1. Do you feel like you are distracted for example when speaking with someone or reading a magazine?

- 4 Very often
- 3 Often
- 2 Sometimes
- 1 Few times
- 0 Never

1. Do you have any difficulties staying in alert and reacting quickly when something you didn’t expect happens? Example, avoiding a car when crossing the street

- 4 Very often
- 3 Often
- 2 Sometimes
- 1 Few times
- 0 Never

1. When the television is on and people around are talking loudly, do you have any difficulties to focus on a particular conversation?

- 4 Very often
- 3 Often
- 2 Sometimes
- 1 Few times
- 0 Never

1. Do you have difficulties to do 2 different things at the same time? Example, having a conversation with someone while watching television, or doing some housekeeping while cooking a lunch on the gas stove

- 4 Very often
- 3 Often
- 2 Sometimes
- 1 Few times
- 0 Never

1. Do you have difficulties to focus on something for more then 20 minutes? Example, listening to the news, reading a magazine, watching a sitcom, attending a school lesson

- 4 Very often
- 3 Often
- 2 Sometimes
- 1 Few times
- 0 Never

1. Do you have difficulties planning something in advance? Example, updating your health care card, getting some money from your post office account, or planning how to spend your budget for the month?

- 4 Very often
- 3 Often
- 2 Sometimes
- 1 Few times
- 0 Never

1. Do you have difficulties to organize your daily activities? Such as shopping, cooking, cleaning the house, fixing stuff, doing some laundry…

- 4 Very often
- 3 Often
- 2 Sometimes
- 1 Few times
- 0 Never

1. Do you have difficulties to change your way of thinking or your manner of doing something the way you’re used to do it when you’re asked to do so and you agree to make these changes?

- 4 Very often
- 3 Often
- 2 Sometimes
- 1 Few times
- 0 Never

1. Do you have difficulties to find your words, to make sentences, to understand the meaning of some words, to pronounce them, to designate objects by their name

- 4 Very often
- 3 Often
- 2 Sometimes
- 1 Few times
- 0 Never

1. Do you have difficulties to do usual activities? Example, to dress up or button a shirt, to introduce a key in a lock, to use a spoon?

- 4 Very often
- 3 Often
- 2 Sometimes
- 1 Few times
- 0 Never
