# Supplementary material for: The Self-Assessment Scale of Cognitive Complaints in Schizophrenia: A validation study in Tunisian population
Source: BMC Psychiatry. 2009 Oct 8;9:66. doi: 10.1186/1471-244X-9-66 (PMC2766383; doi:10.1186/1471-244X-9-66)
Supplement: Additional file 3 — Table S1: demographic characteristics and psychiatry history of pre-test sample. this table describes the sociodemographic characteristics of pre-test sample as well as its psychiatric history. [file 1471-244X-9-66-S3.DOC]

Additional file 3

**TableS1**: demographic characteristics and psychiatry history of pre-test sample

| Variable |  |  |
| --- | --- | --- |
| Age (years; mean, SD) | 34 | 8.9 |
| Gender (n)  Male  Female | 35  3 |  |
| Years of education (mean,SD) | 9 | 3.3 |
| Marital status (n)  Single  Married | 35  3 | 86.8%  13.2% |
| Occupation (n)  Unemployed  Working  Studying  Retired | 25  11  1  1 | 65.8%  29%  2.6%  2.6% |
| Duration of illness (years; mean, SD) | 10.34 | 6.89 |
| Number of hospitalisations (mean, min-max) | 2.76 | [0-20] |
| Total period of hospital stay (months; mean, min-max) | 2.38 | [0-20] |
| Neuroleptics (n)  First generation  Second generation | 32  6 | 84.2 %  15.8 % |
