# Supplementary material for: The Self-Assessment Scale of Cognitive Complaints in Schizophrenia: A validation study in Tunisian population
Source: BMC Psychiatry. 2009 Oct 8;9:66. doi: 10.1186/1471-244X-9-66 (PMC2766383; doi:10.1186/1471-244X-9-66)
Supplement: Additional file 4 — examples for items 13, 15, 18 and 19 of the SASCCS. these are the examples that the investigator could provide to the patient when administering the SASCCS to clarify the meaning of items 13, 15, 18 and 19. [file 1471-244X-9-66-S4.DOC]

Additional file 4

For item 13, the investigator could give to the patient the example of being on alert when lunch is cooking on the gas stove and he had to quickly turn off the gas when he would smell burning.

For item 15, the investigator could give as an example: watching a program on TV and eating at the same time; talking on the cell phone while driving.

For item 18, the investigator could give to the patient, if he was a student, the example of organizing daily activities such as attending school lessons, doing some sports, visiting friends, meeting the doctor… If the patient was an adult, the investigator could provide him with the following examples: going to work/going to the job centre, doing some shopping, doing some housekeeping…

For item 19, the investigator could give to the patient, if he was a student, the example of being asked by the teacher to change his wrong strategy while trying to resolve a mathematical problem. If the patient was an adult, the investigator could him give the example of a professional situation such as changing his wrong way of sorting files when he was asked to do it by his chief.
